# Meta-epidemiology

**DOI:** 10.4178/epih/e2014019

**Published:** 2014-09-25

**Authors:** Jong-Myon Bae

**Affiliations:** Department of Preventive Medicine, Jeju National University School of Medicine, JeJu, Korea

**Keywords:** Review literature as topic, Meta-analysis as topic, Bias, Epidemiology

## Abstract

The concept of meta-epidemiology has been introduced with considering the methodological limitations of systematic review for intervention trials. The paradigm of meta-epidemiology has shifted from a statistical method into a new methodology to close gaps between evidence and practice. Main interest of meta-epidemiology is to control potential biases in previous quantitative systematic reviews and draw appropriate evidences for establishing evidence-base guidelines. Nowadays, the network meta-epidemiology was suggested in order to overcome some limitations of meta-epidemiology. To activate meta-epidemiologic studies, implementation of tools for risk of bias and reporting guidelines such as the Consolidated Standards for Reporting Trials (CONSORT) should be done.

## INTRODUCTION

To establish the best decision-making processes in healthcare service, new fields of study including evidence-based medicine, evidence-based guidelines, and evidence-based health policy have emerged [1]. In addition, for academic cultivation, a new research methodology called systematic reviews (SR) was proposed, under which, the existing generated evidences are systematically collected and evaluated for synthesis into more valid and useful evidence [2-5].

In particular, the success of SR methodology in resolving the controversy over administering beta-blockers in myocardial infarction patients [6] has firmly established its use in published original articles (OA) related to randomized-controlled clinical trials (RCTs) investigating the efficacy of new medicinal or procedural interventions [7,8].

However, given the fact that the subject of SR is OA, some arguments have shown that, even in the development stage, SR methodology cannot overcome inherent limitations of OAs [4, 9-13]. Noble [5] summarized existing suggestions about the advantages and disadvantages of SRs. As one of the breakthroughs for overcoming these limitations, a new terminology, meta-epidemiology, was introduced [14]. In light of this trend, this study aimed to investigate the background of emergence, definition, purposes and research outcomes from its practical applications of meta-epidemiology.

## INTRODUCTION TO META-EPIDEMIOLOGY

### Emerging background of meta-epidemiology

While studies using SR methodology were being actively published, some of these studies with the same research hypotheses began to yield conflicting results; moreover, additional problems were identified in the SR reasoning owing to critical limitations inherent in the OA itself [15-19]. Problems associated with errors that can occur while performing the SR research process, include heterogeneity [20,21], publication bias [9,22-24], and outcome reporting bias [25-27], among others; however, studies have shown that fundamental problems in the methodology associated with conducting RCT research, such as allocation concealment or post-allocation patient blinding, made it difficult to provide a rationale for SR results [18,28-35].

On the basis of these findings, attempts have been made to evaluate the quality of the OA more strictly when conducting a SR and to apply the meta-analysis upon appraising the results of specific items [30,36-39]. In particular, as concept separation and establishment for allocation concealment and post-allocation blinding took place, entering the year 2000 [40,41], a serious movement was seen to confirm the validity of study results from SRs on RCT studies that did not reflect these in the research plan [18,27,30,37,42-45]. With this background, diverse methods, such as mega-regression [4,46-48], imputation [15], informative missing odds ratio [26,49], two statistical models [33], and others were attempted and the term “meta-epidemiology” was introduced [14,16,36,50].

### Definition and purpose of meta-epidemiology

According to Zhang [14], the term “meta-epidemiology” first appeared in published literature in 1997, in an editorial review by Naylor [50]. In 2002, Sterne et al. [16] attempted to make its meaning more explicit by referring to it as a “statistical method” for examining the influence of qualitative problems in RCTs. However, it is now in the process of being recognized as another epidemiological research methodology that controls meta-confounders, similar to traditional epidemiological research methodology that controls confounding variables [14,30,51]. Here, the difference from traditional epidemiology is that the subjects of traditional epidemiological studies are individuals, whereas those of meta-epidemiological studies are OAs that published the results of RCTs performed.

Thus, meta-epidemiology is based on the combination of two concepts: epidemiology and meta-analysis. To fit the purposes of these two concepts, meta-epidemiology strives to achieve the following: (1) to describe the distribution of research evidence for a specific question; (2) to examine heterogeneity and associated risk factors; and (3) to control bias across studies and summarize research evidence as appropriate [14].

### Meta-epidemiology in the literature

Journals that have been published to date with the word “meta-epidemiology” in the journal title or research methodology section have been organized by year as shown in the Appendix 1. Published results with earnest applications of meta-epidemiology are seen after 2008, whereas initial studies were performed to control the influence of allocation concealment and post-allocation blinding [30,32]. As an example, Wood et al. [30] took 1,346 clinical trial papers, which were subjects in 146 published meta-analyses, and divided them, based on the existence or non-existence of allocation concealment and post-allocation blinding, and then re-analyzed them. It was shown that when these items were not properly followed, the subjective evaluations of their effects were exaggerated. More recently, a trend of applying potential meta-confounders, such as genotype [52], study design [36,53], and the number of participants [54], can be seen.

### Meta-meta-epidemiology and network meta-epidemiology

As seen in the aforementioned background of meta-epidemiology, if meta-epidemiology was developed to control diverse SR results, then the word “meta-meta-epidemiology” can be proposed for diverse results from meta-epidemiology [55].

Nonetheless, meta-epidemiology has a few limitations [47,55, 56]. First, the study results that allow analysis are dichotomous and cannot handle continuous outcomes; second, with the reduced number of journals as study subjects, statistical power is limited; and third, indirect comparisons cannot be applied. With the goal of overcoming these limitations, Chaimani et al. [47] proposed the term “network meta-epidemiology.” Concurrently, the term “mixed treatment comparison meta-analysis” was introduced to emphasize the point about making (in)direct comparisons when multiple intervention types are introduced [7,46]. To execute this, the development of research conducting tools [57,58], Copas parametric model [59,60], graphs presented [61], and published items [62], are currently under way.

For easily distinguishing the concepts of meta-meta-epidemiology and network meta-epidemiology, derived from meta-epidemiology as seen above, Trinquart et al. [55] presented a mutual comparison table.

## CONCLUSIONS AND SUGGESTIONS

Valid SR results must be present to develop good clinical diagnostic guidelines, which would ultimately contribute to the improvement of overall healthcare. This is the background and aim of the meta-epidemiology emergence. To obtain valid SR results, the key challenge is to improve the quality of RCTs that are the subject of analysis by SR [36,63]. To this end, some suggestions are being made.

First, development and distribution of standardized quality assessment tools is needed to accurately assess the risk of error occurrence [64]. Cochrane Collaboration has proposed a tool called risk of bias [65-67]; more active meta-epidemiologic studies are needed using such tools to investigate what influences are imposed on SR reasoning [36].

Second, there is a need to more clearly organize the concepts behind the terminologies used in RCT quality assessment [68]. This is because concepts such as allocation concealment and post-allocation blinding must be revised and disseminated in a unified manner to researchers, as well as existing research methodology textbooks.

Third, in order to accurately verify and interpret the RCT study results in SRs, reports must be made without overlooking any of the designated items [35]. Since there are some reports indicating that following the reporting guideline in The Consolidated Standards for Reporting Trials (CONSORT) improves the quality of journals [69,70], RCT researches would need to obey this guideline.

Fourth, there is an international movement to have the study registered in an open venue prior to conducting the study to prevent overlooking study results, because cases of conflicting between initial plans and final results were surfaced [71]. As there is also the advantage of reducing publication bias [24], there is a need to accept this wholeheartedly in accordance with the international trend.

## Appendix 1. Examples of meta-epidemiologic studies

**Table:**

Meta-confounders	References
Concealment & blindness	A01
Placebo control vs. untreated control	A02
Concealment & blindness	A03
Genetic polymorphism	A04
Exclusion of patients	A05
Randomization & effect size	A06
Single center vs. multicenter	A07
Concealment	A08
Experimental vs. observational design	A09
Study design	A10
Sample size	A11
