# Supplementary material for: Meta-epidemiology
Source: Epidemiol Health. 2014 Sep 25;36:e2014019. doi: 10.4178/epih/e2014019 (PMC4220603; doi:10.4178/epih/e2014019)
Supplement: Supplementary file 1 [file epih-36-e2014019-supplementary.pdf]

<방법론>

메타역학

Meta-Epidemiology

Epidemiology and Health

Abstract

The concept of meta-epidemiology has been introduced with considering the methodological limitations of systematic review for intervention trials. The paradigm of meta-epidemiology has shifted from a statistical method into a new methodology to close gaps between evidence and practice. Main interest of meta-epidemiology is to control potential biases in previous quantitative systematic reviews and draw appropriate evidences for establishing evidence-base guidelines. Nowadays, the network meta-epidemiolgy was suggested in order to overcome some limitations of meta-epidemiology. To activate meta-epidemiologic studies, implementation of tools for risk of bias and reporting guidelines such as the Consolidated Standards for Reporting Trials (CONSORT) should be done.

Key words: Review literature as topic, Meta-analysis as topic, Bias, Epidemiology

Epidemiology and Health

1. 서론

보건의료서비스 제공에 있어 최선의 의사결정 판단을 하려는 움직임은 근거중심의학 (Evidence-based medicine), 근거중심 지침 (Evidence-based guideline), 근거중심 보건정책 (Evidence-based Health Policy) 등의 새로운 학문 영역들이 등장하게 되었다 [1]. 그리고 학문적 개척을 위하여 기존에 만들어진 (generation) 근거들을 체계적으로 수집하고 평가하여, 보다 타당하고 유용한 근거로 합성 (synthesis)하려는 움직임에 체계적 고찰 (systematic review, SR)란 새로운 연구방법론이 제시되었다 [2-5].

특히 심근경색환자에 있어 베타차단체 투여 여부의 결정에 있어 SR 방법론을 적용하여 논란을 잠재운 경험을 하면서 [6], 현재는 약물 혹은 시술 등의 새로운 개입 (intervention)의 효능 (efficacy)를 알아보는 무작위배정 비교임상시험 (Randomized-controlled clinical trials, RCT)과 관련하여 발표한 원 논문 (original article, OA)들에 관한 체계적 고찰 연구방법론은 확립된 상황이다 [7,8].

그러나 메타분석의 연구대상은 OA라는 점에서, OA가 갖는 근본적인 한계 때문에 SR 방법론으로 이를 극복하는 데는 한계가 있다는 주장이 개발 당시부터 제기되어 왔다 [4, 9-13]. SR을 둘러싼 그동안의 장단점에 대한 주장들을 Noble [5]은 정리해서 제시하였다. 이런 한계들을 극복하려는 돌파구의 하나로 메타역학 (Meta-Epidemiology)이란 신조어가 제시된 것이다 [14]. 이런 흐름에 있어 메타역학이 대두한 배경, 정의와 목적, 그리고 실제 적용한 연구 성과들을 살펴보고자 한다.

2. 본론: 메타역학의 개론

Epidemiology and Health

가. 메타역학 대두 배경

SR을 적용한 논문들이 활발히 발표되는 가운데, 같은 연구가설을 가진 SR들 간에도 상이한 결과가 도출되거나, OA 자체가 가진 치명적 한계로 SR 추론에 대한 문제가 제기되는 상황이 연출되기 시작하였다 [15-19]. SR 연구 수행 과정에서 발생될 수 있는 오류들인 이질성 (heterogeneity) 통제 [20,21], 출판 오류 (publication bias) [9, 22-24], 결과보고 오류 (outcome reporting bias) [25-27] 등도 문제가 되겠지만, RCT 연구 수행상 문제가 되는 대상자 배정과정의 숨김 (allocation concealment) 이나 배정후 환자의 눈가림법 (blinding) 등의 근본적인 방법론상 문제로 인하여 SR 결과 추론을 어렵게 만든다는 주장들이 제기되어 왔다 [18, 28-35].

이에 따라 SR 수행시 연구대상이 된 OA의 질적 수준을 보다 엄격히 평가해서, 특정 항목의 평가 결과에 따라 메타분석을 달리 적용해 보려는 시도들이 있었다 [30, 36-39]. 특히 배정과정 숨김과 배정후 눈가림법이 2000년을 들어서면서 개념 분리와 정립이 이루어지면서 [40,41], 계획서에 이를 반영하지 않고 수행된 RCT들을 연구대상으로 삼은 SR에 있어 연구결과의 타당성을 확인하려는 움직임이 본격화되었다 [18, 27, 30, 37, 42-45]. 이런 배경에서 mega-regression [4, 46-48], imputation [15], Informative missing odds ratio [26, 49], two statistical model [33] 등의 다양한 시도들이 있었으며, 메타역학이란 용어가 제기된 것이다 [14, 16, 36, 50].

Epidemiology and Health

나. 메타역학의 정의와 목적

Zhang [14]에 따르면, 메타역학이란 용어가 문헌에 처음으로 등장한 것은 1997년 David Naylor의 편집자 논평이라고 한다 [50]. 2002년 Sterne et al [16] 이 RCT의 질적 문제

가 어떤 영향을 미치는 가를 알아보기 위한 '통계학적 방법'이라고 한정적인 의미를 부여하였다. 그러나 지금은 교란변수들을 통제하는 전통 역학연구방법론처럼, 메타 교란변수(meta-confoudners)를 통제하는 또 하나의 역학연구방법론으로 정립하고 있는 중에 있다 [14, 30, 51]. 여기서 전통역학과의 차이점이라면, 기존의 전통역학의 연구 대상이 개별 사람이라면, 메타역학의 연구대상은 RCT 수행결과를 발표한 OA인 것이다.

메타역학은 epidemiology와 meta-analysis 의 두 개념이 합쳐 만들어 진 것으로, 이들의 목적에 맞추어 메타역학은 다음 3가지를 지향하고 있다 [14]. (1) 특정 연구가설에 따른 근거들의 분포를 살펴보며 (to describe distribution of research evidences for a specific question) (2) 이질성 여부와 이를 야기시킨 요소들을 알아내고 (to examine heterogeneity and associated risk factors) (3) 연구들 간의 개입된 오류들을 확인 통제하여 최적화된 종합적 근거를 도출하려는 것이다 (to control bias across studies and summarize research evidence as appropriate).

Epidemiology and Health

다. 메타역학의 실제 적용 사례

지금까지 논문 제목이나, 연구방법론에 메타역학이란 용어를 제시하고 발표한 논문들을 연도별로 정리한 것이 Appendix 이다. 본격적으로 메타역학을 적용한 결과 발표는 2008년 이후이며, 초기에는 배정과정 숨김과 배정 후 눈가림법의 영향을 통제하려는 연구가 수행되었다 [30, 32]. 그 한 예로 Wood et al. [30]는 기존에 발표된 146개의 메타분석 대상인 1,346개 임상시험 논문들을 배정과정 숨김과 배정 후 눈가림법 여부에 따라 다시 나누어 재 분석을 했을 때, 이들의 항목이 제대로 지켜지지 않은 경우에서 주관적 효과 평가가 과장됨을 보여주었다. 최근으로 올수록 유전자형 [52], 연구설계 [36, 53], 대상자 수 [54]

같이 다양한 잠재적인 메타교란변수들 (potential meta-confounders)을 적용하고 있는 흐름을 알 수 있다.

라. 메타메타역학 (meta-meta-epidemiology)과 망메타역학 (Network meta-epidemiology)

앞서 메타역학이 제기된 배경에서 보듯이, 다양한 SR 결과를 제어하기 위하여 메타역학이 생겨났다면, 메타역학의 결과가 다양하다면 이를 제어하기 위하여 메타-메타역학이란 용어가 제기될 수 있다 [55].

한편, 메타역학은 몇 가지 한계를 가지고 있다 [47, 55, 56]. 첫째로 분석이 가능한 연구 결과는 이분형이어서 연속형 결과들을 다룰 수 없으며, 둘째로 대상이 되는 논문들의 숫자가 줄어들어 통계적 검정력에서 제한이 생기고, 셋째로 간접비교 (indirect comparison)을 적용할 수 없다는 것이다. 이런 제한들을 극복하려는 취지에서 Chiamani et al [47]는 망메타역학 (network meta-epidemiology)란 용어를 제시하였다. 특히 개입의 종류를 여러 개로 한 경우의 직간접 비교를 한다는 점을 강조하는 mixed treatment comparison meta-analysis 란 용어로도 제시되었다 [7, 46]. 이를 수행하기 위하여, 연구수행 도구 [57,58], Copas parametric model [59,60], 제시하는 그래프 [61], 발표 항목 [62] 등의 개발 등이 현재 진행 중에 있다.

이상과 같이 메타역학에서 파생한 메타메타역학과 망메타역학의 개념을 잘 구분짓도록 Trinquart et al [55]는 상호비교한 표를 제시하였다.

3. 결어 및 제언

Epidemiology and Health

타당한 SR 결과가 있어야만, 좋은 임상진료지침이 만들어 지며, 결국 보건의료 전체의 질 향상에 기여할 수 있다. 메타역학이 제기된 배경과 지향하는 목적도 바로 이것이다. 올바른 SR 결과 도출을 위해서는 SR의 분석대상이 되는 RCT의 질을 향상시켜야 하는 것이 핵심적인 과제가 된다 [36, 63]. 이를 위하여 몇 가지 제언을 하고 마치고자 한다.

첫째, 오류가 발생할 수 있는 위험을 제대로 평가하는 질 평가 표준 도구 개발 및 보급이 필요하다 [64]. 코크란 연합회에서는 RoB (risk of bias)란 도구를 제시하고 있는데 [65-67], 이러한 도구를 활용하여 SR 추론에 어떤 영향을 미치는가를 알아보는 메타역학 연구의 활성화가 필요하다 [36].

둘째, RCT의 질 평가에 적용하는 용어들의 개념을 보다 분명히 정립할 필요가 있다 [68]. 배정과정 숨기기와 배정후 눈가림법 같은 개념들은 기존의 연구방법 교과서 뿐만 아니라, 연구 수행자들에게도 통일되게 수정 보급되어야 하기 때문이다.

셋째, RCT의 연구결과를 SR에서 제대로 확인하고 해석하기 위해서는 정해진 항목들을 빠뜨리지 않고 보고해야 한다 [35]. The Consolidated Standards for Reporting Trials (CONSORT) 보고 지침에 따를 경우 논문의 질적 향상에 도움이 된다는 보고가 있어 [69,70], RCT 연구자들은 이를 가능한 준수하는 것이 필요하다.

넷째, 애초 계획서와 보고한 결과 보고서와는 차이가 있다는 사실이 부각되면서 [71], 연구수행 직전에 공개적인 곳에 등록하여 연구결과가 누락되지 않도록 하자는 국제적인 움직임이 있다. 출판오류도 줄일 수 있다는 장점 [24]도 있어 세계적 추세에 맞추어 이를 적극

Epidemiology and Health

수용하는 것이 필요하다.

감사의 글

이 논문은 2014학년도 제주대학교 학술진흥연구비 지원사업에 의하여 연구되었음

Epidemiology and Health

References

1. Bae JM, Park BJ, Ahn YO. Perspectives of clinical epidemiology in Korea. J Korean Med Assoc 2013;56:718-723. (korean)

2. Ioannidis JP, Schmid CH, Lau J. Meta-analysis in hematology and oncology. Hematol Oncol Clin North Am 2000;14:973-991.

3. Ahn HS, Kim HJ. An introduction to systematic review. J Korean Med Assoc 2014;57:49-59. (korean)

4. Khoshdel A, Attia J, Carney SL. Basic concepts in meta-analysis: A primer for clinicians. Int J Clin Pract 2006;60:1287-1294.

5. Noble JH Jr. Meta-analysis: Methods, strengths, weaknesses, and political uses. J Lab Clin Med 2006;147:7-20.

6. Freemantle N, Cleland J, Young P, Mason J, Harrison J. beta Blockade after myocardial infarction: systematic review and meta regression analysis. BMJ 1999;318:1730-1737.

7. Salanti G, Higgins JP, Ades AE, Ioannidis JP. Evaluation of networks of randomized trials. Stat Methods Med Res 2008;17:279-301.

8. Bae JM. Methodological issues for Systematic Reviews of Diagnostic Tests Assessments. Epidemiol Health 2014. (in press) (korean)

9. Egger M, Smith GD, Steme JA. Uses and abuses of meta-analysis. Clin Med 2001;1:478-484.

10. Stevens KR, Ledbetter CA. Basics of evidence-based practice. Part 1: The nature of the evidence. Semin Perioper Nurs 2000;9:91-97.

11. Michels KB. Quo vadis meta-analysis? A potentially dangerous tool if used without adequate rules. Important Adv Oncol 1992:243-8.

12. Finckh A, Tramèr MR. Primer: strengths and weaknesses of meta-analysis. Nat Clin Pract Rheumatol 2008;4:146-1452.

13. Morris RD. Meta-analysis in cancer epidemiology. Environ Health Perspect 1994;102 Suppl 8:61-66.

14. Zhang W. Meta-epidemiology: building the bridge from research evidence to clinical practice.

Epidemiology and Health

Osteoarthritis Cartilage 2010;18 Suppl 2:S1.

15. Robertson C, Idris NR, Boyle P. Beyond classical meta-analysis: can inadequately reported studies be included? Drug Discov Today 2004;9:924-931.

16. Sterne JA, Jüni P, Schulz KF, Altman DG, Bartlett C, Egger M. Statistical methods for assessing the influence of study characteristics on treatment effects in 'meta-epidemiological' research. Stat Med 2002;21:1513-1524.

17. Olsen O, Gøtzsche PC. Cochrane review on screening for breast cancer with mammography. Lancet. 2001;358:1340-1342.

18. Schulz KF, Chalmers I, Hayes RJ, Altman DG. Empirical evidence of bias. Dimensions of methodological quality associated with estimates of treatment effects in controlled trials. JAMA 1995;273:408-412.

19. Gluud LL. Bias in clinical intervention research. Am J Epidemiol 2006;163:493-501.

20. Ioannidis JP. Interpretation of tests of heterogeneity and bias in meta-analysis. J Eval Clin Pract 2008;14:951-957.

21. Turner RM, Davey J, Clarke MJ, Thompson SG, Higgins JP. Predicting the extent of heterogeneity in meta-analysis, using empirical data from the Cochrane Database of Systematic Reviews. Int J Epidemiol 2012;41:818-827.

22. Hopewell S, Loudon K, Clarke MJ, Oxman AD, Dickersin K. Publication bias in clinical trials due to statistical significance or direction of trial results. Cochrane Database Syst Rev 2009;(1):MR000006.

23. Mahid SS, Qadan M, Hornung CA, Galandiuk S. Assessment of publication bias for the surgeon scientist. Br J Surg 2008;95:943-949.

24. Thornton A, Lee P. Publication bias in meta-analysis: its causes and consequences. J Clin Epidemiol 2000;53:207-216.

25. Kirkham JJ, Dwan KM, Altman DG, Gamble C, Dodd S, Smyth R, et al. The impact of outcome reporting bias in randomised controlled trials on a cohort of systematic reviews. BMJ 2010;340:c365.

26. White IR, Welton NJ, Wood AM, Ades AE, Higgins JP. Allowing for uncertainty due to missing data in

Epidemiology and Health

meta-analysis--part 2: hierarchical models. Stat Med 2008;27:728-745.

27. Savović J, Jones HE, Altman DG, Harris RJ, Jüni P, Pildal J, et al. Influence of reported study design characteristics on intervention effect estimates from randomized, controlled trials. Ann Intern Med 2012;157:429-438.

28. Jüni P, Altman DG, Egger M. Systematic reviews in health care: Assessing the quality of controlled clinical trials. BMJ 2001;323:42-46.

29. Hróbjartsson A, Thomsen AS, Emanuelsson F, Tendal B, Hilden J, Boutron I, et al. Observer bias in randomised clinical trials with binary outcomes: systematic review of trials with both blinded and non-blinded outcome assessors. BMJ 2012;344:e1119.

30. Wood L, Egger M, Gluud LL, Schulz KF, Jüni P, Altman DG, et al. Empirical evidence of bias in treatment effect estimates in controlled trials with different interventions and outcomes: meta-epidemiological study. BMJ 2008;336:601-605.

31. Nüesch E, Trelle S, Reichenbach S, Rutjes AW, Bürgi E, Scherer M, et al. The effects of excluding patients from the analysis in randomised controlled trials: meta-epidemiological study. BMJ 2009;339:b3244.

32. Nüesch E, Reichenbach S, Trelle S, Rutjes AW, Liewald K, Sterchi R, et al. The importance of allocation concealment and patient blinding in osteoarthritis trials: a meta-epidemiologic study. Arthritis Rheum 2009;61:1633-1641.

33. Siersma V, Als-Nielsen B, Chen W, Hilden J, Gluud LL, Gluud C.. Multivariable modelling for meta-epidemiological assessment of the association between trial quality and treatment effects estimated in randomized clinical trials. Stat Med 2007;26:2745-2758.

34. Montori VM, Bhandari M, Devereaux PJ, Manns BJ, Ghali WA, Guyatt GH. In the dark: the reporting of blinding status in randomized controlled trials. J Clin Epidemiol 2002;55:787-790.

35. Kjaergard LL, Villumsen J, Gluud C. Reported methodologic quality and discrepancies between large and small randomized trials in meta-analyses. Ann Intern Med 2001;135:982-989.

36. Savović J, Jones H, Altman D, Harris R, Jűni P, Pildal J, et al. Influence of reported study design

Epidemiology and Health

characteristics on intervention effect estimates from randomised controlled trials: combined analysis of meta-epidemiological studies. Health Technol Assess 2012;16:1-82.

37. Moher D, Pham B, Jones A, Cook DJ, Jadad AR, Moher M, et al. Does quality of reports of randomised trials affect estimates of intervention efficacy reported in meta-analyses? Lancet 1998;352:609-613.

38. Balk EM, Bonis PA, Moskowitz H, Schmid CH, Ioannidis JP, Wang C, et al. Correlation of quality measures with estimates of treatment effect in meta-analyses of randomized controlled trials. JAMA 2002;287:2973-2982.

39. Jackson R, Ameratunga S, Broad J, Connor J, Lethaby A, Robb G, et al. The GATE frame: critical appraisal with pictures. Evid Based Med 2006;11:35-38.

40. Altman DG, Schulz KF. Statistics notes: Concealing treatment allocation in randomised trials. BMJ 2001;323:446-447.

41. Pildal J, Chan AW, Hróbjartsson A, Forfang E, Altman DG, Gøtzsche PC. Comparison of descriptions of allocation concealment in trial protocols and the published reports: cohort study. BMJ 2005;330:1049.

42. Pildal J, Hróbjartsson A, Jørgensen KJ, Hilden J, Altman DG, Gøtzsche PC. Impact of allocation concealment on conclusions drawn from meta-analyses of randomized trials. Int J Epidemiol 2007;36:847-857.

43. Schulz KF. Assessing allocation concealment and blinding in randomized controlled trials: why bother? ACP J Club 2000;132:A11-A12.

44. Hróbjartsson A, Pildal J, Chan AW, Haahr MT, Altman DG, Gøtzsche PC. Reporting on blinding in trial protocols and corresponding publications was often inadequate but rarely contradictory. J Clin Epidemiol 2009;62:967-973.

45. Egger M, Juni P, Bartlett C, Holenstein F, Sterne J. How important are comprehensive literature searches and the assessment of trial quality in systematic reviews? Empirical study. Health Technol Assess 2003;7:1-76.

46. Salanti G, Dias S, Welton NJ, Ades AE, Golfinopoulos V, Kyrgiou M, et al. Evaluating novel agent effects in

Epidemiology and Health

multiple-treatments meta-regression. Stat Med 2010;29:2369-2383.

47. Chaimani A, Vasiliadis HS, Pandis N, Schmid CH, Welton NJ, Salanti G. Effects of study precision and risk of bias in networks of interventions: a network meta-epidemiological study. Int J Epidemiol 2013;42:1120-1131.

48. Rücker G, Carpenter JR, Schwarzer G. Detecting and adjusting for small-study effects in meta-analysis. Biom J 2011;53:351-368.

49. Lyles RH, Allen AS, Dana Flanders W, Kupper LL, Christensen DL. Inference for case-control studies when exposure status is both informatively missing and misclassified. Stat Med 2006;25:4065-4080.

50. Naylor CD. Meta-analysis and the meta-epidemiology of clinical research. BMJ 1997;315:617-619.

51. Le Lorier J, Grégoire G. Meta-analysis and the meta-epidemiology of clinical research. Comments on paper by author of editorial were unwarranted. BMJ 1998;316:311-312.

52. Valdes AM, Arden NK, Tamm A, Kisand K, Doherty S, Pola E, et al. A meta-analysis of interleukin-6 promoter polymorphisms on risk of hip and knee osteoarthritis. Osteoarthritis Cartilage 2010;18:699-704.

53. Tzoulaki I, Siontis KC, Ioannidis JP. Prognostic effect size of cardiovascular biomarkers in datasets from observational studies versus randomised trials: meta-epidemiology study. BMJ 2011;343:d6829.

54. Dechartres A, Trinquart L, Boutron I, Ravaud P. Influence of trial sample size on treatment effect estimates: meta-epidemiological study. BMJ 2013;346:f2304.

55. Trinquart L, Dechartres A, Ravaud P. Commentary: Meta-epidemiology, meta-meta-epidemiology or network meta-epidemiology? Int J Epidemiol 2013;42:1131-1133.

56. Veroniki AA, Vasiliadis HS, Higgins JP, Salanti G. Evaluation of inconsistency in networks of interventions. Int J Epidemiol 2013;42:332-345.

57. Salanti G, Del Giovane C, Chaimani A, Caldwell DM, Higgins JP. Evaluating the quality of evidence from a network meta-analysis. PLoS One 2014;9:e99682.

58. Hutton B, Salanti G, Chaimani A, Caldwell DM, Schmid C, Thorlund K, et al. The quality of reporting methods and results in network meta-analyses: an overview of reviews and suggestions for improvement.

Epidemiology and Health

PLoS One 2014;9:e92508.

59. Mavridis D, Sutton A, Cipriani A, Salanti G. A fully Bayesian application of the Copas selection model for publication bias extended to network meta-analysis. Stat Med 2013;32:51-66.

60. Salanti G, Marinho V, Higgins JP. A case study of multiple-treatments meta-analysis demonstrates that covariates should be considered. J Clin Epidemiol 2009;62:857-864.

61. Chaimani A1 Higgins JP, Mavridis D, Spyridonos P, Salanti G. Graphical tools for network meta-analysis in STATA. PLoS One 2013;8:e76654.

62. Bafeta A, Trinquart L, Seror R, Ravaud P. Analysis of the systematic reviews process in reports of network meta-analyses: methodological systematic review. BMJ 2013;347:f3675.

63. De Vito C, Manzoli L, Marzuillo C, Anastasi D, Boccia A, Villari P. A systematic review evaluating the potential for bias and the methodological quality of meta-analyses in vaccinology. Vaccine 2007;25:8794-8806.

64. Moja LP, Telaro E, D'Amico R, Moschetti I, Coe L, Liberati A. Assessment of methodological quality of primary studies by systematic reviews: results of the metaquality cross sectional study. BMJ 2005;330:1053.

65. Hartling L, Ospina M, Liang Y, Dryden DM, Hooton N, Krebs Seida J, et al. Risk of bias versus quality assessment of randomised controlled trials: cross sectional study. BMJ 2009;339:b4012.

66. Higgins JP, Altman DG, Gøtzsche PC, Jüni P, Moher D, Oxman AD, et al. The Cochrane Collaboration's tool for assessing risk of bias in randomised trials. BMJ 2011;343:d5928.

67. Schouten LM, Hulscher ME, van Everdingen JJ, Huijsman R, Grol RP. Evidence for the impact of quality improvement collaboratives: systematic review. BMJ 2008;336:1491-1494.

68. Devereaux PJ, Manns BJ, Ghali WA, Quan H, Lacchetti C, Montori VM, et al. Physician interpretations and textbook definitions of blinding terminology in randomized controlled trials. JAMA 2001;285:2000-2003.

69. Augestad KM, Berntsen G, Lassen K, Bellika JG, Wootton R, Lindsetmo RO, et al. Standards for reporting randomized controlled trials in medical informatics: a systematic review of CONSORT adherence in RCTs on clinical decision support.. J Am Med Inform Assoc 2012;19:13-21.

Epidemiology and Health

70. Hopewell S, Dutton S, Yu LM, Chan AW, Altman DG. The quality of reports of randomised trials in 2000 and 2006: comparative study of articles indexed in PubMed. BMJ 2010;340:c723.

71. Hill CL, LaValley MP, Felson DT. Discrepancy between published report and actual conduct of randomized clinical trials. J Clin Epidemiol 2002;55:783-786.

Epidemiology and Health

Appendix. Examples of meta-epidemiologic studies

Studies*	Meta-confounders
A01 (2008)	concealment & blindness
A02 (2008)	placebo control vs untreated control
A03 (2009)	concealment & blindness
A04 (2009)	genetic polymorphism
A05 (2009)	exclusion of patients
A06 (2010)	randomization & effect size
A07 (2011)	single center vs multicenter
A08 (2011)	concealment
A09 (2011)	experimental vs observational design
A10 (2012)	study design
A11 (2013)	sample size

* Reference number (publication year)

Epidemiology and Health

A01. Wood L, Egger M, Gluud LL, Schulz KF, Jüni P, Altman DG, et al. Empirical evidence of bias in treatment effect estimates in controlled trials with different interventions and outcomes: meta-epidemiological study. BMJ 2008;336:601-605.

A02. Zhang W, Robertson J, Jones AC, Dieppe PA, Doherty M. The placebo effect and its determinants in osteoarthritis: meta-analysis of randomised controlled trials. Ann Rheum Dis 2008;67:1716-1723.

A03. Nüesch E, Reichenbach S, Trelle S, Rutjes AW, Liewald K, Sterchi R, et al. The importance of allocation concealment and patient blinding in osteoarthritis trials: a meta-epidemiologic study. Arthritis Rheum

2009;61:1633-1641.

A04. Valdes AM, Arden NK, Tamm A, Kisand K, Doherty S, Pola E, et al. A meta-analysis of interleukin-6 promoter polymorphisms on risk of hip and knee osteoarthritis. Osteoarthritis Cartilage 2010;18:699-704.

A05. Nüesch E, Trelle S, Reichenbach S, Rutjes AW, Bürgi E, Scherer M, et al. The effects of excluding patients from the analysis in randomised controlled trials: meta-epidemiological study. BMJ 2009;339:b3244.

A06. Oliver S, Bagnall AM, Thomas J, Shepherd J, Sowden A, White I, et al. Randomised controlled trials for policy interventions: a review of reviews and meta-regression. Health Technol Assess 2010;14:1-165.

A07. Dechartres A, Boutron I, Trinquart L, Charles P, Ravaud P. Single-center trials show larger treatment effects than multicenter trials: evidence from a meta-epidemiologic study. Ann Intern Med 2011;155:39-51.

A08. Herbison P, Hay-Smith J, Gillespie WJ. Different methods of allocation to groups in randomized trials are associated with different levels of bias. A meta-epidemiological study. J Clin Epidemiol 2011;64:1070-1075.

A09. Tzoulaki I, Siontis KC, Ioannidis JP. Prognostic effect size of cardiovascular biomarkers in datasets from observational studies versus randomised trials: meta-epidemiology study. BMJ 2011;343:d6829.

A10. Savović J, Jones H, Altman D, Harris R, Jűni P, Pildal J, et al. Influence of reported study design characteristics on intervention effect estimates from randomised controlled trials: combined analysis of meta-epidemiological studies. Health Technol Assess 2012;16:1-82.

A11. Dechartres A, Trinquart L, Boutron I, Ravaud P. Influence of trial sample size on treatment effect estimates: meta-epidemiological study. BMJ 2013;346:f2304.

Epidemiology and Health

초록

약물이나 시술 등의 개입에 관한 효과를 알아보는 임상시험을 대상으로 체계적 고찰을 수행하는 과정에서 한계점을 극복하기 위하여 '메타역학'이란 개념이 만들어졌다. 처음에는 통계적 방법론에서 시작했으나 지금은 근거-진료간의 간격을 메꾸는 새로운 연구방법론으로 자리잡게 되었다. 메타역학 방법론의 주된 목적은 기존의 체계적 고찰 결과의 오류를 알아보고, 근거바탕의 진료지침을 수립하는데 필요한 최적의 근거를 제공하는 것이다. 그리고 메타역학의 한계점을 뛰어넘기 위하여 '망 메타역학'이란 개념도 제시되었다. 국내 메타역학 연구의 활성화를 도모하기 위하여 메타분석 개념을 소개하고 발전 방안들을 제시하였다.

Epidemiology and Health
